# John Broom

**Published:** 1900-06

**Authors:** 


John Broom died at his residence on February 26th, in his fifty-eighth year, after a short illness. The deceased gentleman received his medical education at the medical schools of Liverpool, Sheffield, and the London Hospital, and qualified as L.R.C.P. Irel., and L.R.C.S. Edin. in 1870, three years later graduating as M.D. of Brussels University with distinction. Dr. Broom shortly afterwards commenced practice in Sheffield, and in 1877 came to Clifton as partner with the late Dr. T. E. Clark, the partnership terminating in 1879.

